# Supplementary figures and images for: Cbfβ regulates Wnt/β-catenin, Hippo/Yap, and Tgfβ signaling pathways in articular cartilage homeostasis and protects from ACLT surgery-induced osteoarthritis
Source: eLife. 2024 May 28;13:e95640. doi: 10.7554/eLife.95640 (PMC11132684; doi:10.7554/eLife.95640)

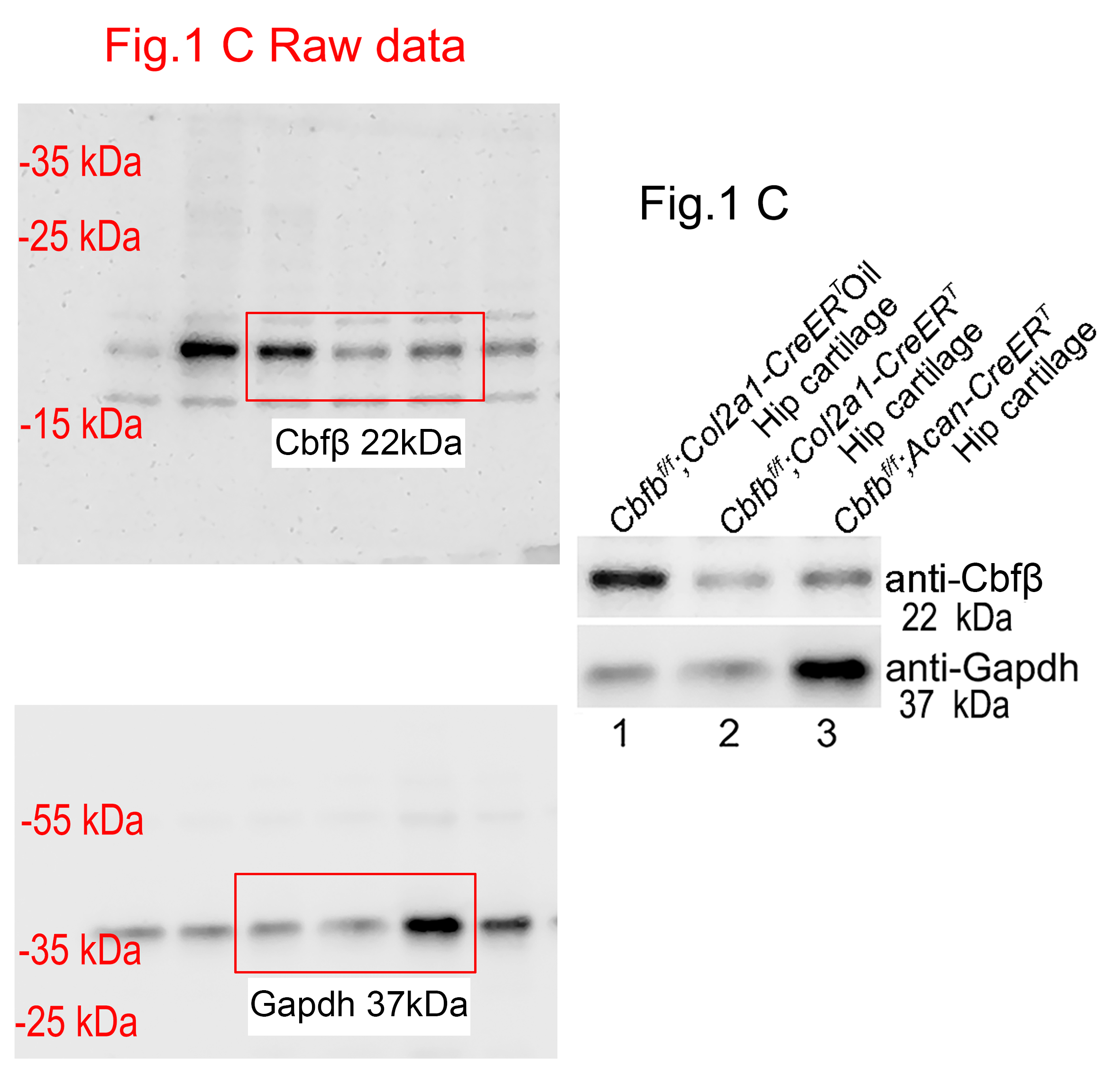

Supplement: Figure 1—source data 1. [file elife-95640-fig1-data1.zip › fig 1 source data 1/fig 1 source data 1.tif]

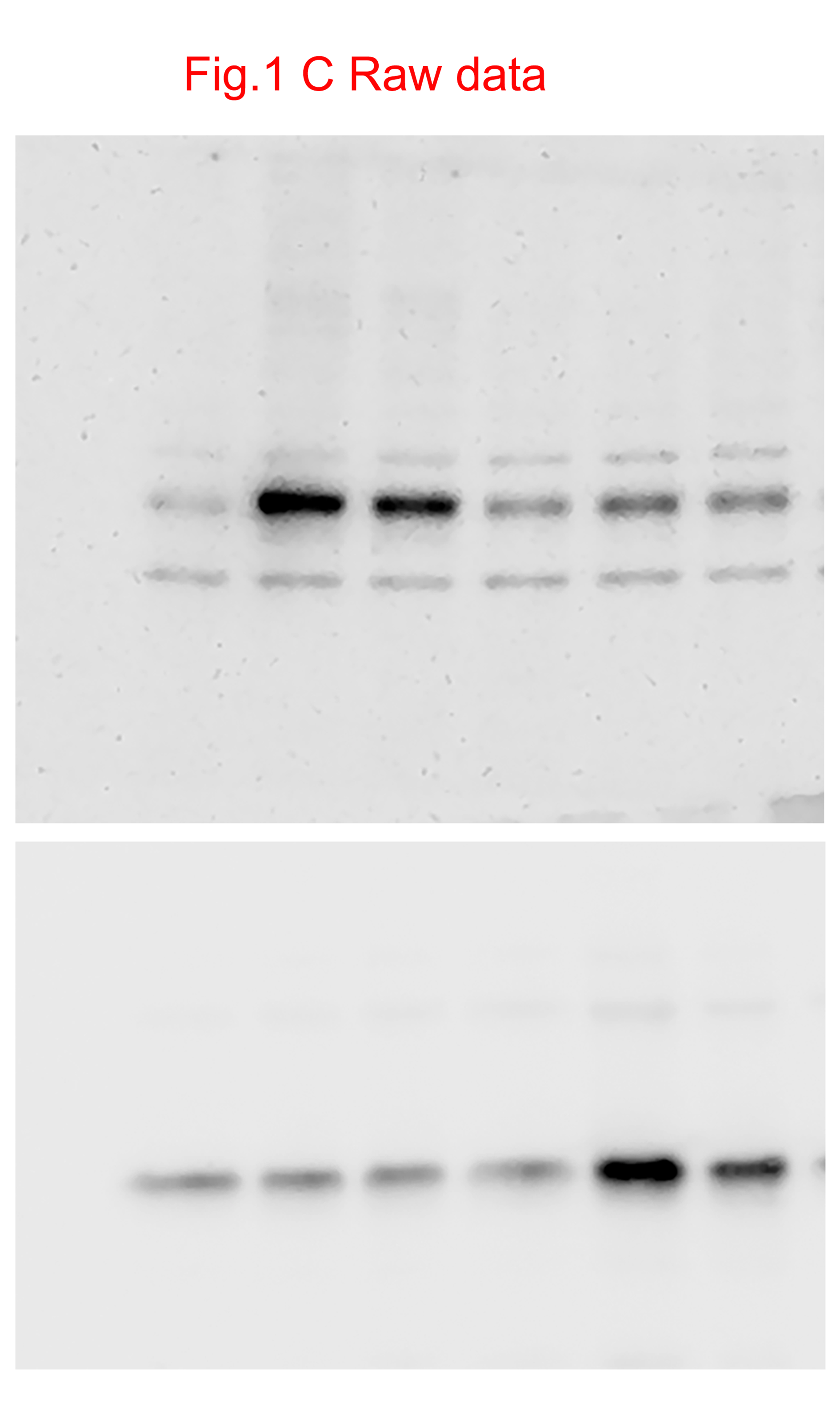

Supplement: Figure 1—source data 2. [file elife-95640-fig1-data2.zip › fig 1 source data 2.tif]

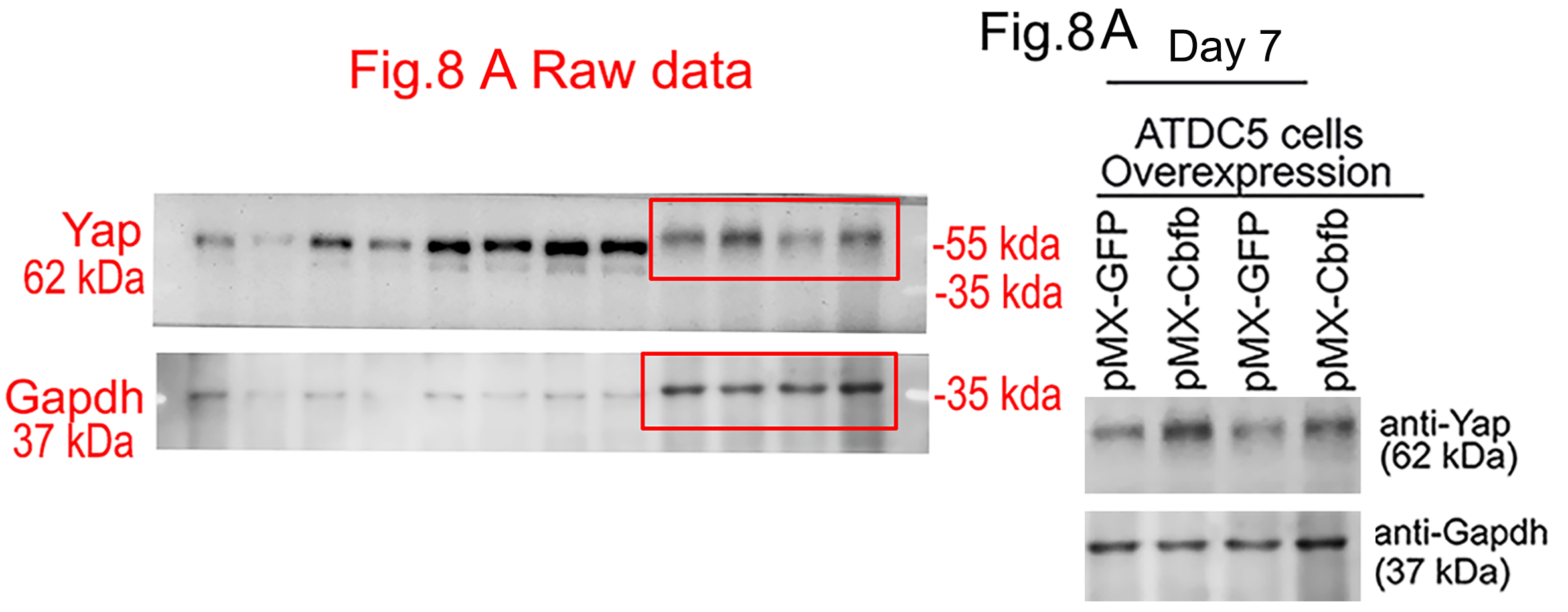

Supplement: Figure 8—source data 1. [file elife-95640-fig8-data1.zip › fig.8 source data 1.tif]

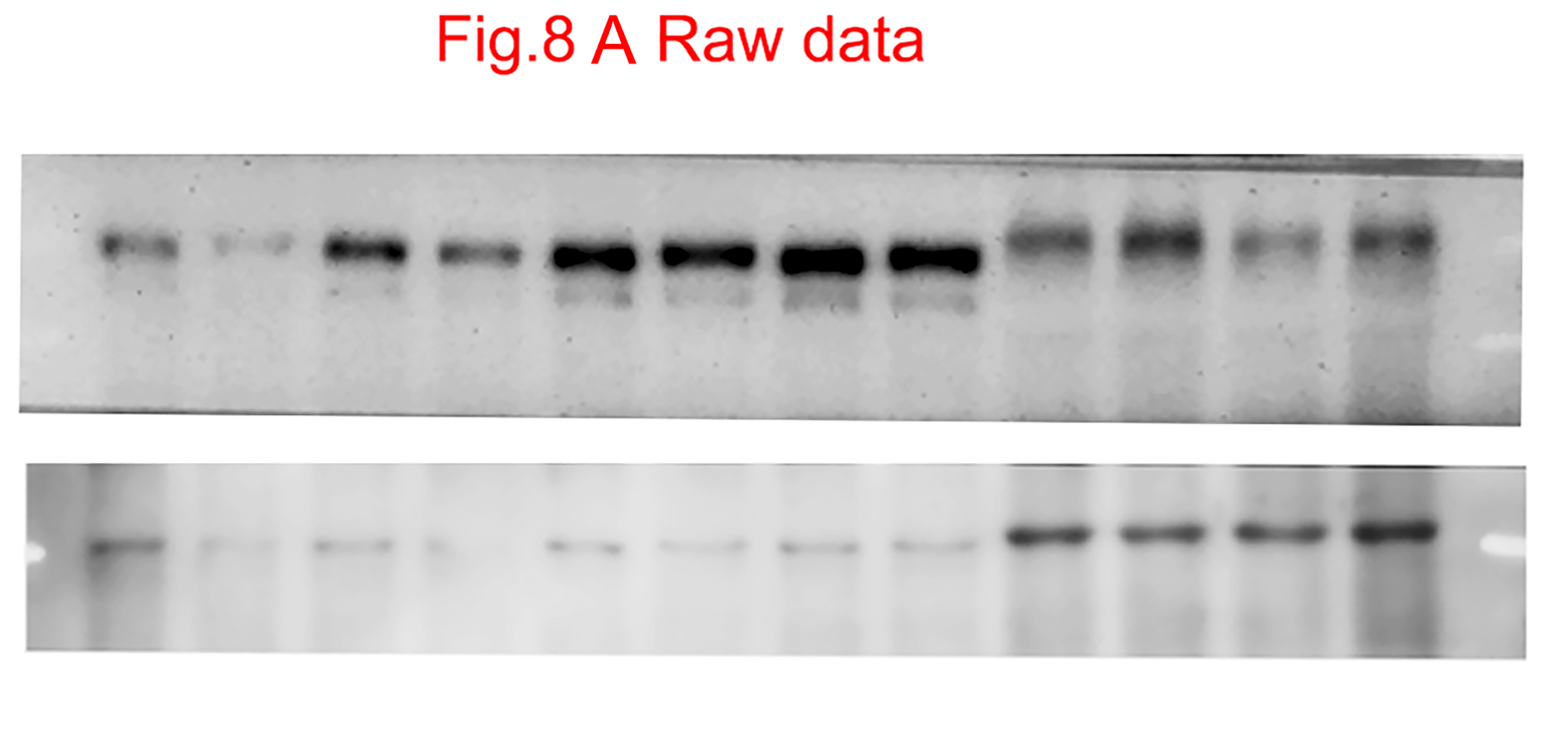

Supplement: Figure 8—source data 2. [file elife-95640-fig8-data2.zip › fig.8 source data 2.tif]

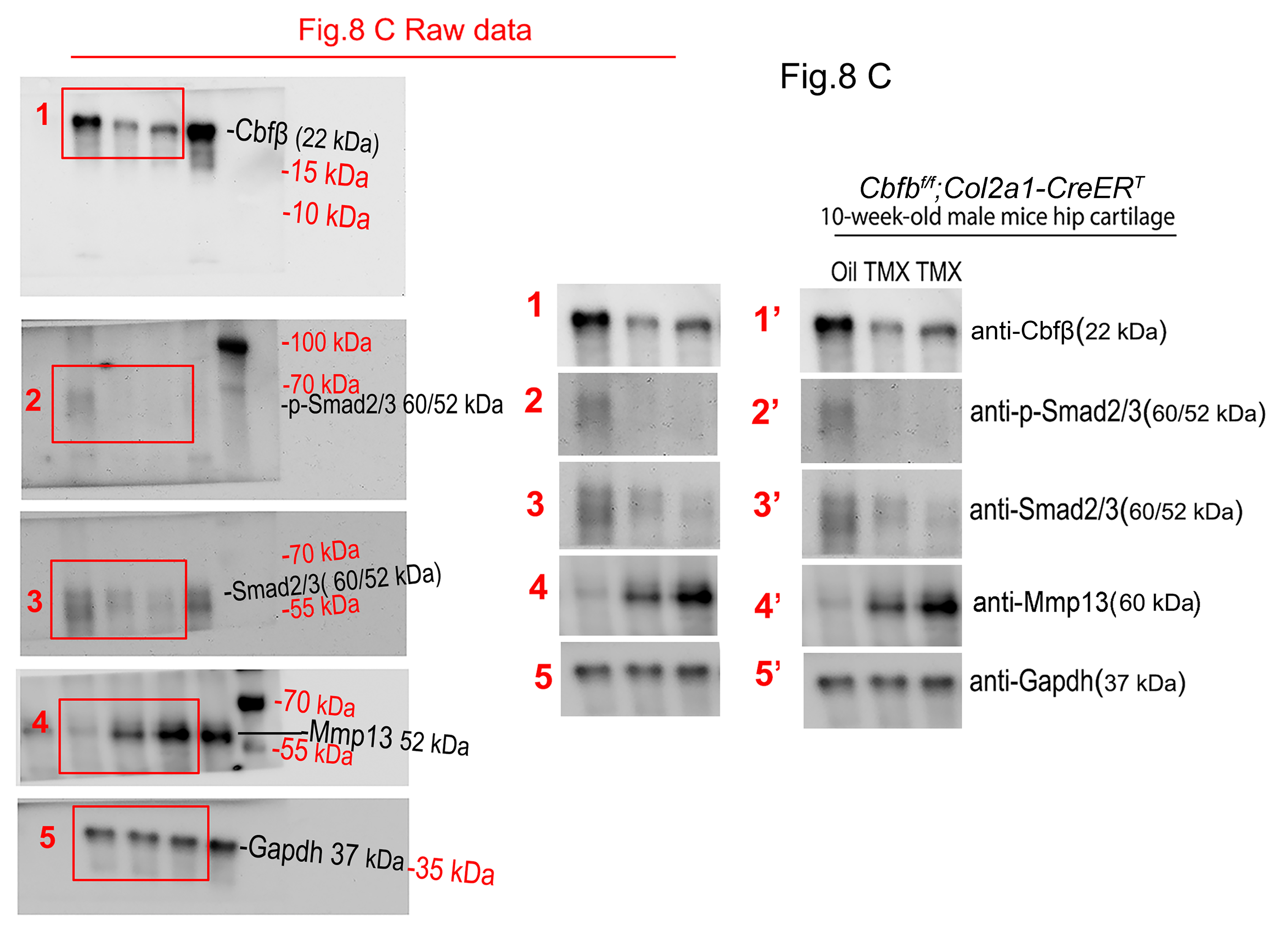

Supplement: Figure 8—source data 3. [file elife-95640-fig8-data3.zip › fig 8 source data 3/fig.8 source data 3.tif]

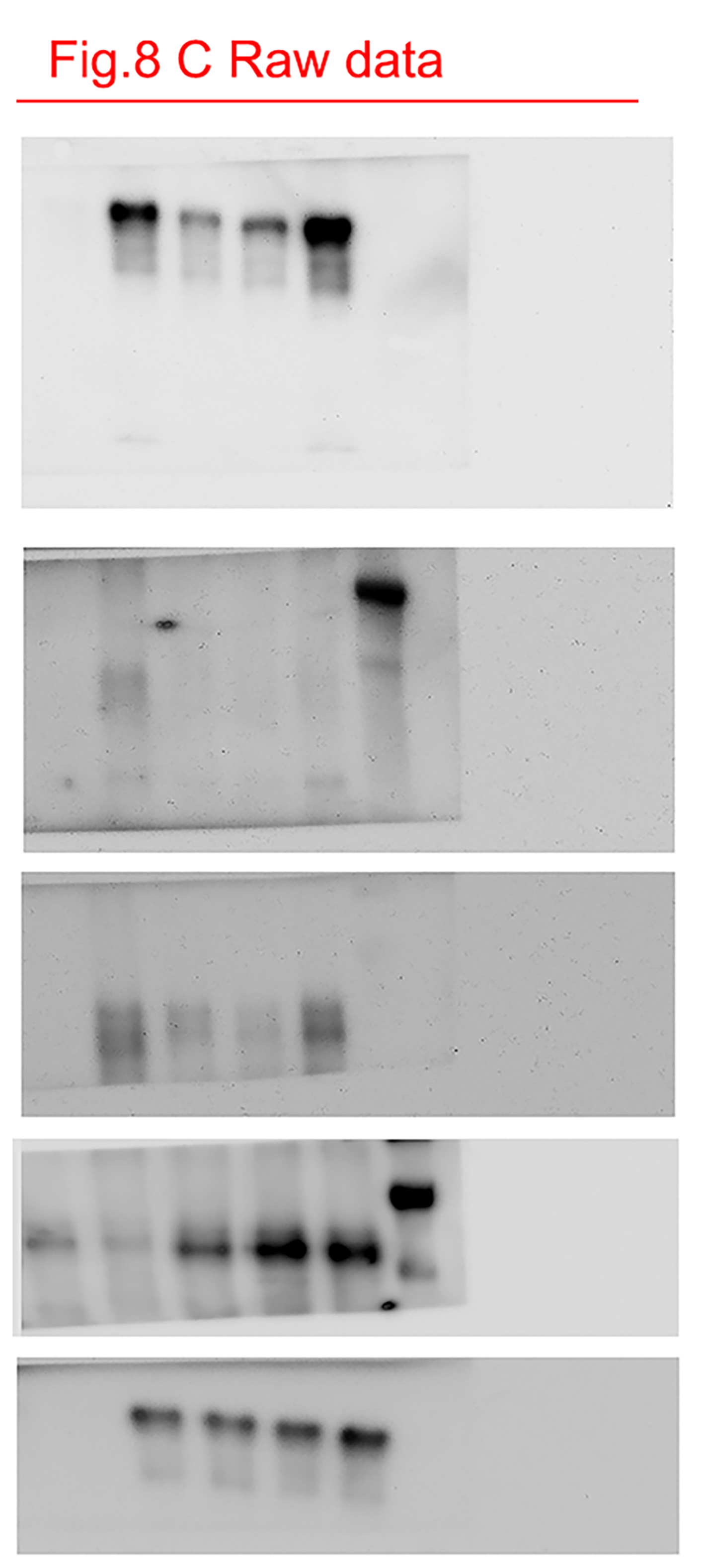

Supplement: Figure 8—source data 4. [file elife-95640-fig8-data4.zip › fig.8 source data 4.tif]

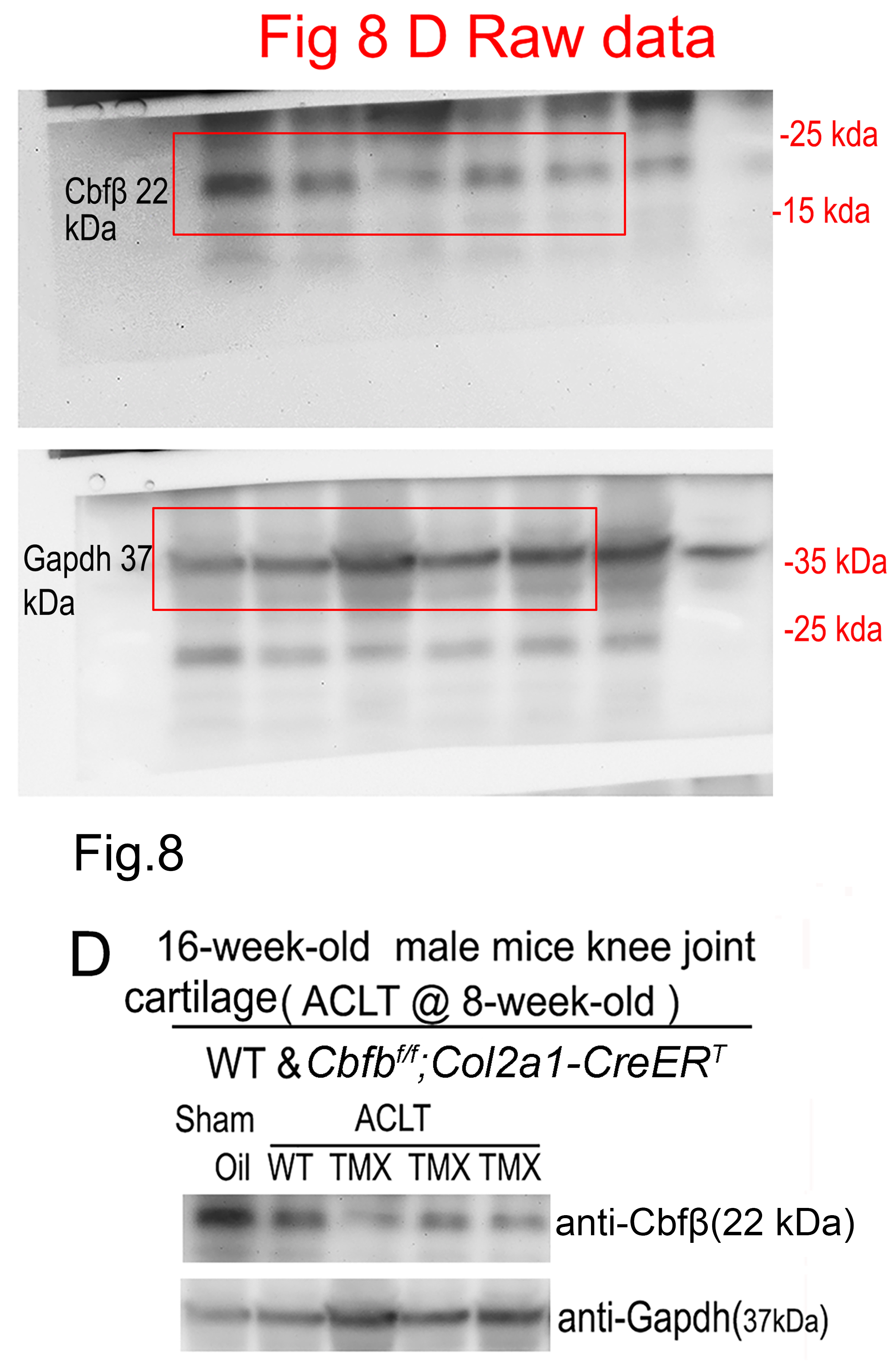

Supplement: Figure 8—source data 5. [file elife-95640-fig8-data5.zip › fig 8 source data 5/fig.8 source data 5.tif]

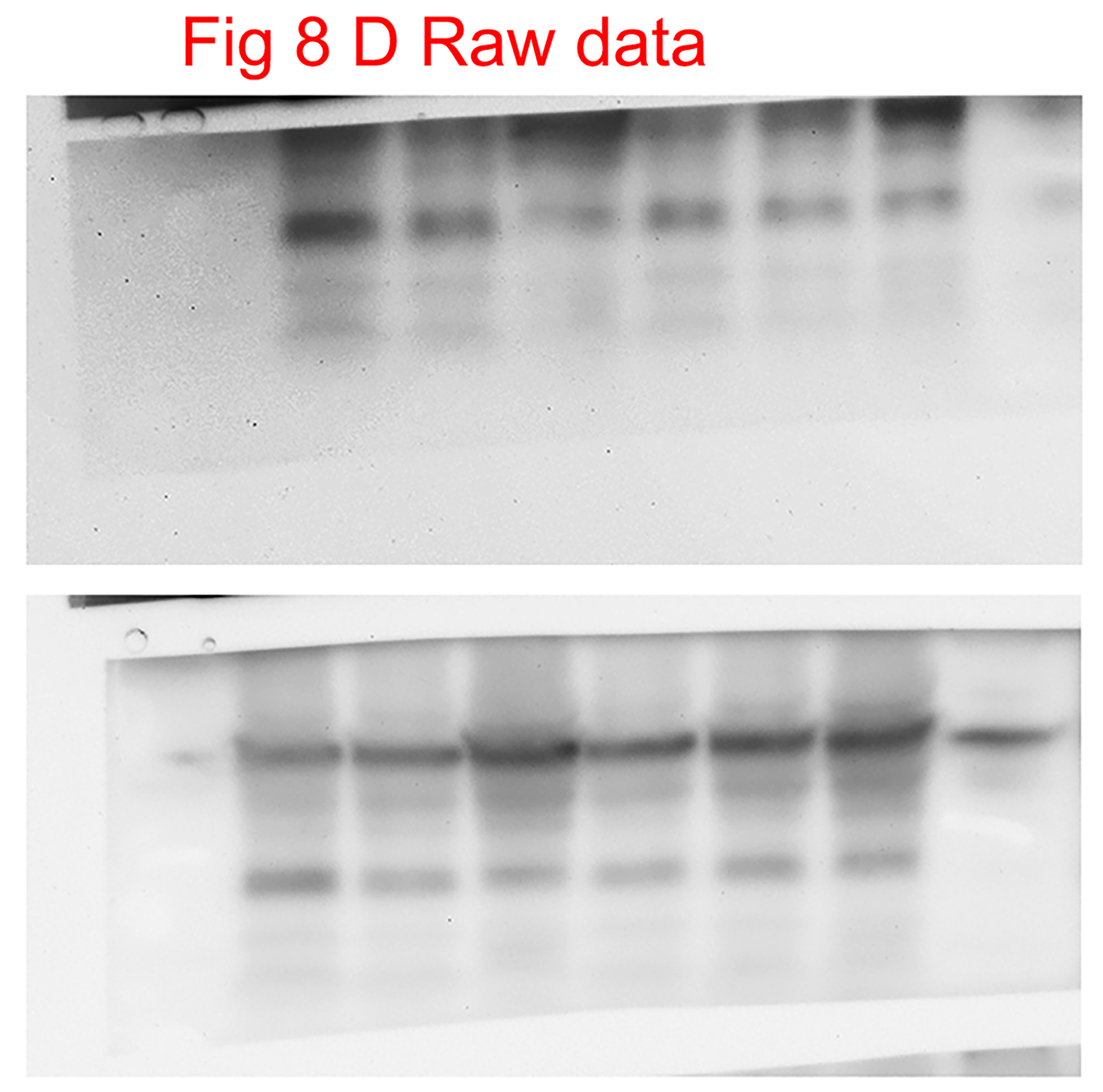

Supplement: Figure 8—source data 6. [file elife-95640-fig8-data6.zip › fig.8 source data 6.tif]

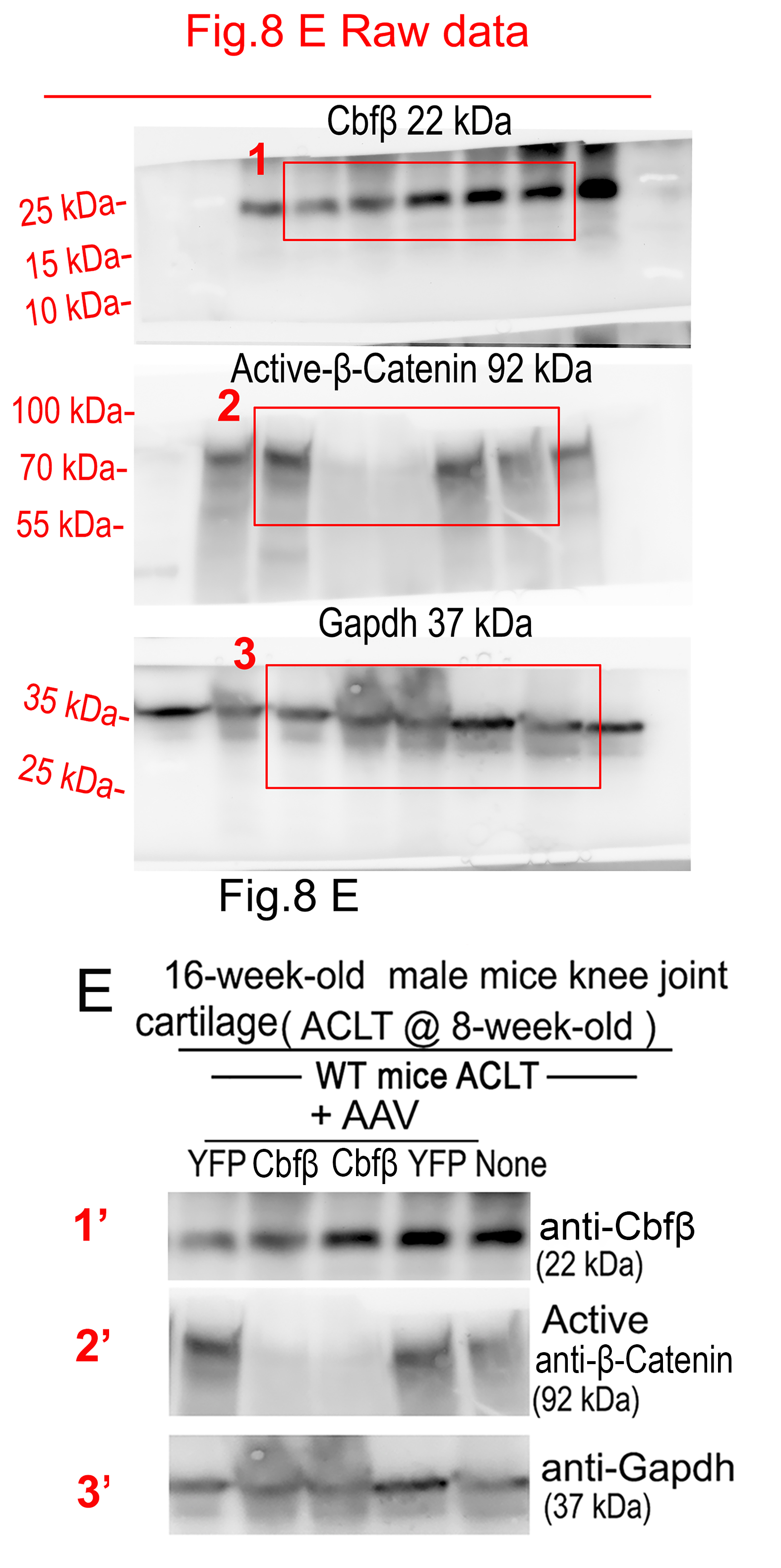

Supplement: Figure 8—source data 7. [file elife-95640-fig8-data7.zip › fig 8 source data 7/fig.8 source data 7.tif]

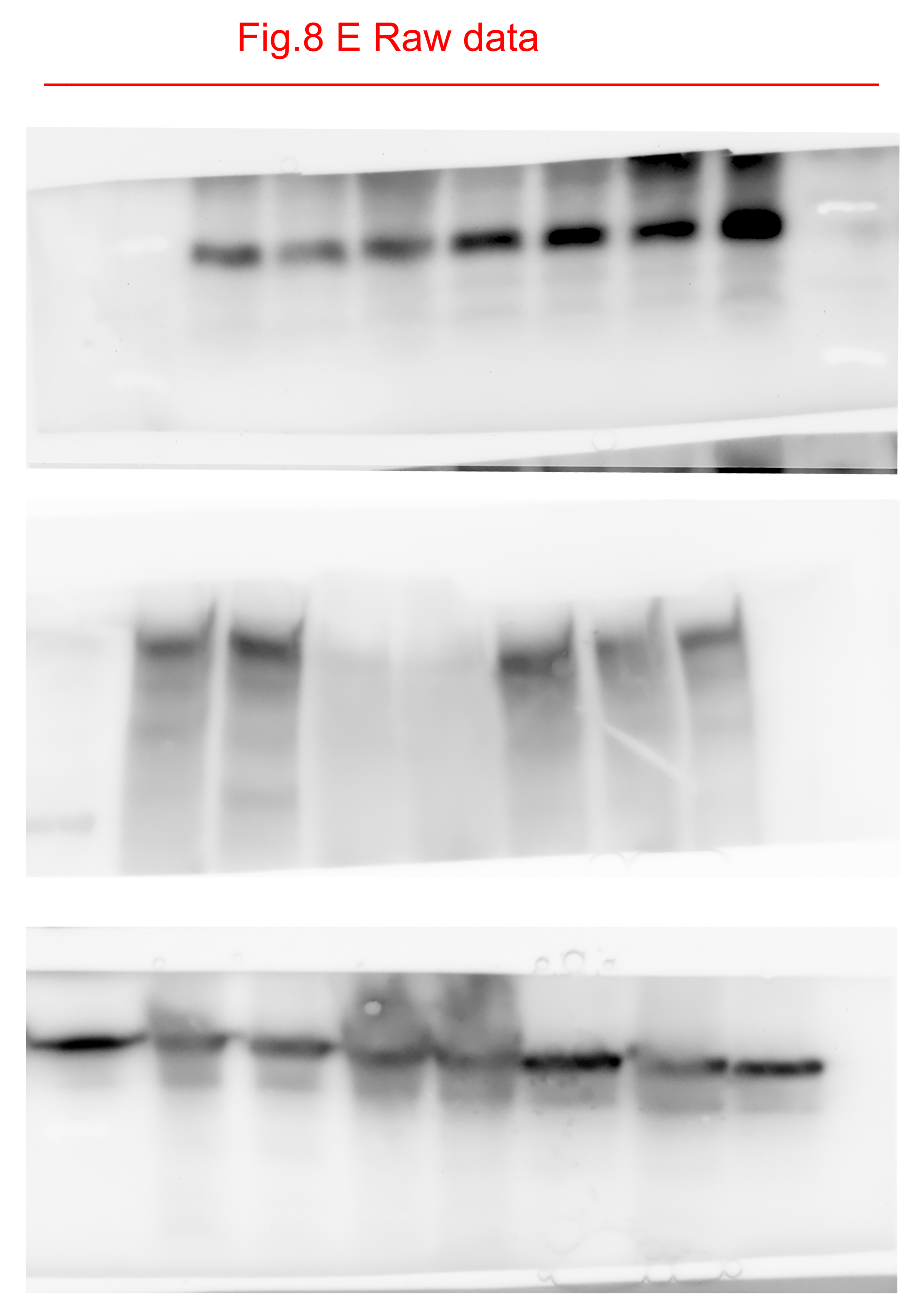

Supplement: Figure 8—source data 8. [file elife-95640-fig8-data8.zip › fig.8 source data 8.tif]
